# Effects of Pioglitazone Mediated Activation of PPAR-γ on CIDEC and Obesity Related Changes in Mice

**DOI:** 10.1371/journal.pone.0106992

**Published:** 2014-09-11

**Authors:** Bilal Haider Shamsi, Chaofeng Ma, Saima Naqvi, Yanfeng Xiao

**Affiliations:** 1 Pediatrics Department, 2nd Affiliated Hospital of Medical College of Xi'an Jiaotong University, Xi'an City, Shaanxi Province, P.R. China; 2 Pathology Department, The Center for Disease Control and Prevention (CDC), Xi'an City, Shaanxi Province, P.R. China; 3 Endocrinology Department, 1st Affiliated Hospital of Medical College of Xi'an Jiaotong University, Xi'an City, Shaanxi Province, P.R. China; Bambino Gesu' Children Hospital, Italy

## Abstract

**Objective:**

Obesity is a metabolic disorder that can lead to high blood pressure, increased blood cholesterol and triglycerides, insulin resistance, and diabetes mellitus. The aim was to study the effects of pioglitazone mediated sensitization of peroxisome proliferator-activated receptor gamma (PPAR-γ) on the relationship of Cell death-inducing DFFA-like effector C (CIDEC) with obesity related changes in mice.

**Methods:**

Sixty C57B/L6 mice weighing 10–12g at 3 weeks of age were randomly divided into 3 groups. Mice in Group 1 were fed on normal diet (ND) while Group 2 mice were given high fat diet (HFD), and Group 3 mice were given high fat diet and treated with Pioglitazone (HFD+P). Body weight, length and level of blood sugar were measured weekly. Quantitative real-time PCR, fluorescence microscopy, and ELISA were performed to analyze the expression of CIDEC and PPAR-γ in visceral adipose tissue (VAT) and subcutaneous adipose tissue (SAT).

**Results:**

Body weight and length of mice increased gradually with time in all groups. Blood sugar in HFD mice started to increase significantly from the mid of late phase of obesity while pioglitazone attenuated blood sugar level in HFD+P mice. The mRNA expressions and protein levels of PPAR-γ and CIDEC genes started to increase in HFD mice as compared to ND mice and decreased gradually during the late phase of obesity in VAT. Pioglitazone enhanced the expression of PPAR-γ and CIDEC genes in HFD+P mice even during the late phase of obesity.

**Conclusion:**

It is insinuated that VAT is associated with late phase obesity CIDEC decrease and insulin resistance, while pioglitazone enhances CIDEC through activation of PPAR-γ, increases its expression, and decreases lipolysis, hence preventing an increase of blood sugar in mice exposed to HFD.

## Introduction

Obesity is one of the key risk factors for many chronic diseases, including diabetes, cardiovascular diseases and cancer [1]. It is recognized as a global epidemic by the World Health Organization (WHO) [2]. Heredity is one of the strong components involved in obesity, but genetic pathways contributing to obesity have not yet been elucidated [3]. Although obesity is a major risk factor for type 2 diabetes mellitus (DM 2) [4], relationship between early and late phases of obesity and increased blood sugar is variable in the literature [5]. Link between insulin resistance, hypertension and abnormal lipid profile is well known and a significant association has been reported between obesity and insulin resistance in young adulthood [6]. Accumulation of lipid droplets in the late phase of obesity followed by lipolysis and increased levels of free fatty acids, damage the insulin signaling and lead towards insulin resistance and DM 2 [7]. It is well documented that adipose tissue does not only function passively to store fat, but is an endocrine organ secreting several biologically active peptides. Altered profile of adipokines secreted by visceral fat in Ames dwarf mice may act as a key contributor to increased insulin sensitivity. Insulin sensitivity was enhanced after removal of visceral fat in normal mice and opposite effect was found in mice with targeted deletion of the growth hormone receptor (GHRKO mice) [8], [9], indicating an important role of visceral fat in regulating insulin action.

High fat diet has been demonstrated to enhance lipogenesis and inhibit apoptosis in adipocytes [10]. However, the mechanisms underlying anti-apoptotic and lipid accumulating effects are still unclear [11]. Cell death-inducing DFFA-like effector C (CIDEC), a member of the CIDE family, is closely related to obesity [12]. Increased expression of CIDEC has been demonstrated in adipogenesis [13], however the role of CIDEC in adipolysis and insulin resistance in the late phase of obesity has not yet been fully discovered [14]. PPAR-γ gene encodes a protein which acts as a regulator of adipocyte differentiation and has been implicated with the pathology of many diseases including obesity, diabetes, atherosclerosis and cancer [15]. The expression of CIDEC/FSP27 in adipocytes is mediated directly or indirectly by PPAR-γ expression [16]. Pioglitazone is an insulin sensitizer that acts through the nuclear receptor PPAR-γ, modulating the transcription of the insulin-sensitive genes and is highly effective oral medication for type 2 diabetes. However, the risks of fluid retention, weight gain, bone loss and congestive heart failure has also been reported from its use [17].

Present research was designed to observe different phases of high fat diet induced obesity and to study the effects of PPAR-γ activator pioglitazone on the relationship of CIDEC in visceral and subcutaneous adipose tissue and other obesity related changes in mice. Pioglitazone helps to maintain the expression of PPAR-γ during late phase of obesity, thus effects CIDEC gene expression and blood sugar.

## Materials and Methods

### Ethics Statement

This study was carried out in strict accordance with the recommendations in the Guide for the Care and Use of Laboratory Animals of the National Institutes of Health. The protocol was approved by the Committee on the Ethics of Animal Experiments of the Xi'an Jiaotong University. All surgery was performed using intra-peritoneal chloroform injection anesthesia, and all efforts were made to minimize suffering.

### Animals

Sixty C57B/L6 mice weighing 10–12 g at 3 weeks of age were obtained and maintained at animal breeding center of Medical College of Xi'an Jiaotong University. Mice had no other associated co morbidity i.e. physical disability and other disease condition. The animal rooms were controlled for temperature (23∼28°C), humidity (55±5%) and light (12 h light-dark cycles). Mice were randomly divided into 3 groups (20 mice in each group). Mice in Group 1 were fed with normal diet (ND) while mice in Group 2 were given high fat diet (HFD) and mice in Group 3 were given high fat diet plus PPAR-γ agonist, pioglitazone (HFD+P). High fat diet contained 45% fat, 13.2% protein and 41.8% carbohydrates [18].

### Body Weight, Length and Blood Sugar

The body weight, body length and six hours fasting blood sugar were measured weekly. Blood sugar was measured with Life Scan Accu-Check glucometer. Body mass index was calculated accordingly with units converted to Kg/m^2^.

### Adipose Tissue Collection

Five mice from each group were anesthetized using intra-peritoneal chloroform injection and sacrificed at 4, 8, 12 and 16 weeks to evaluate the effects of pioglitazone on the expression of CIDEC and PPAR-γ genes during early and late phases of obesity. Visceral adipose tissue (VAT) and Subcutaneous adipose tissue (SAT) were collected and immediately stored in liquid nitrogen at −80°C until further analysis [19].

### Quantitative Real-Time PCR

Total RNAs were isolated from fat tissue of mice using TRIZOL method. RNA was quantified by measuring the optical density (CECIL Instrument, England). Total cellular RNA was reverse transcribed by reverse transcription (RT) PCR according to the manufacture's protocol (RevertAid, Fermentas Life Sciences, International INC, Canada). Resulting cDNAs were subjected to Real-time PCR with SYBR Premix Ex Tag II (TaKaRa, Japan) for the quantitation of CIDEC and PPAR-γ, using primer pairs given in table 1. The amplification program consisted of one cycle of 95°C for 5 minutes, 40 cycles of 95°C for 10 seconds, 55°C for 15 seconds and 72°C for 50 seconds, and finally one cycle of 95°C for 15 seconds. β-actin was used as reference gene.

**Table 1:** Primers used for the genes enumeration by Real time RT-PCR.

Name	Sequence	Annealing Temp
CIDEC-F	TGTCGTGTTAGCACCGCAGAT	55°C
CIDEC-R	CTCCAGCACCAGGGAGAAGG	
PPAR-γ -F	CATGGTGCCTTCGCTGAT	55°C
PPAR-γ –R	CAATGGCCATGAGGGAGTTA	

### Fluorescence Microscopy

Immunofluorescence staining was used to analyze the CIDEC and PPAR-γ distribution on the fatty cells according to already reported protocol. The fat tissue was embedded in the cryo tissue embedding compound and the rabbit sourced diluted antibody with fluorescein isothiocyanate labeled mouse against rabbit IgG antibody, were used. The results were observed under fluorescence microscope [20].

### Level of Protein of CIDEC and PPAR-γ

Adipose fat tissues (VAT and SAT) homogenates were used to determine the level of CIDEC and PPAR-γ by ELISA with commercially available kit purchased from Global Biotech, Shanghai, China, following the instruction of the manufacturer. After stopping the reaction the color was measured as OD units 450 nm on ELISA reader (Bio-Red). The concentration was expressed in ng/ml by using standard curve.

### Statistical analysis

All the data was expressed as Mean ±SD and analyzed by One way ANOVA followed by Bonferroni's post hoc test by Graph pad Prism5 statistical software. Differences among groups were considered statistically significant at P value<0.05.

## Results

### Body length and weight of mice

**Figure 1:**
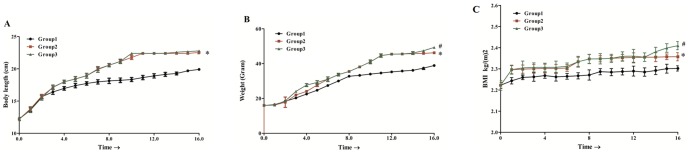
Length, weight and BMI with time, of mice fed on high fat diet (HFD). Mice were divided into three groups: Group1; mice were given normal diet (ND), Group2; mice were given high fat diet (HFD), Group3; mice were given high fat diet and treated with pioglitazone (HFD+P). Weight and length were measured weekly. (A) represents body weight of mice in Group1, Group2 and Group3. (B) represents body length of mice in Group1, Group2 and Group3. (C) represents BMI of mice in Group1, Group2 and Group3. Data was expressed as Mean±SD. *and #, P<0.05, represent significant difference between Group1 and Group2, and Group2 and Group3 respectively.

Body length of mice increased gradually with time in each group which was significantly faster in group 2 (HFD) and group 3 (HFD+P) as compared to mice in group1 (ND). No difference was found in body length of mice between group 2 and group 3 (Fig.1A). The body weight of mice increased during the early phase and maintained the rate of weight gain during the late phase of obesity in all groups. More weight gain was observed in group 3 during mid of early phase and end of late phase of obesity when compared with mice in group 2. Statistically significant difference was observed in weight between group 2 and group 3 when compared with group1 (Fig1B). Increase in BMI was observed in group 2 and group 3 as compared to group1 while significant increase was found in group 3 during second half of late phase of obesity as compared with group 2 (Fig 1C).

### Blood sugar

**Figure 2:**
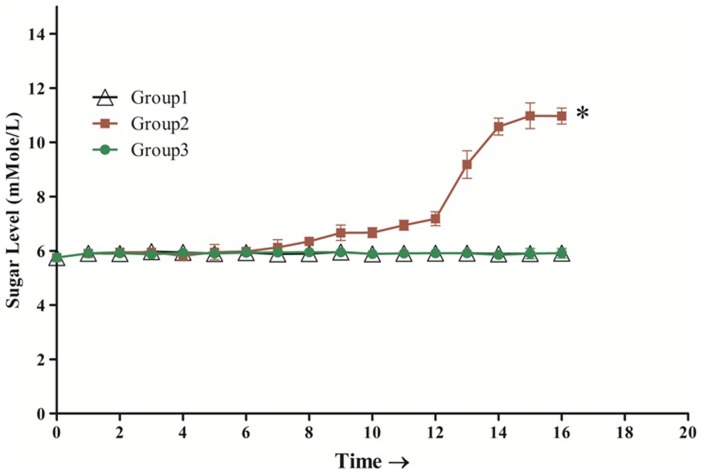
Fasting blood sugar levels in mice. Six hours fasting blood sugar levels were measured weekly with Life Scan Accu-Check Glucometer in all groups of mice. Group1; mice were given normal diet (ND), Group2; mice were given high fat diet (HFD), Group3; mice were given high fat diet and treated with pioglitazone (HFD+P). Data was expressed as Mean±SD. *P<0.05 represents significant difference between two groups.

The blood sugar level remained fairly constant in mice of group 1 and increased significantly in group 2 as compared to group 1. In group 2, sugar level remained constant during the early phase of obesity, increased from mid of the late phase of obesity and remained high in second half of the late phase. The pioglitazone significantly suppressed the blood sugar level in mice of group 3 as compared to group 2 (Fig.2).

### mRNA Expression of CIDEC and PPAR-γ

**Figure 3:**
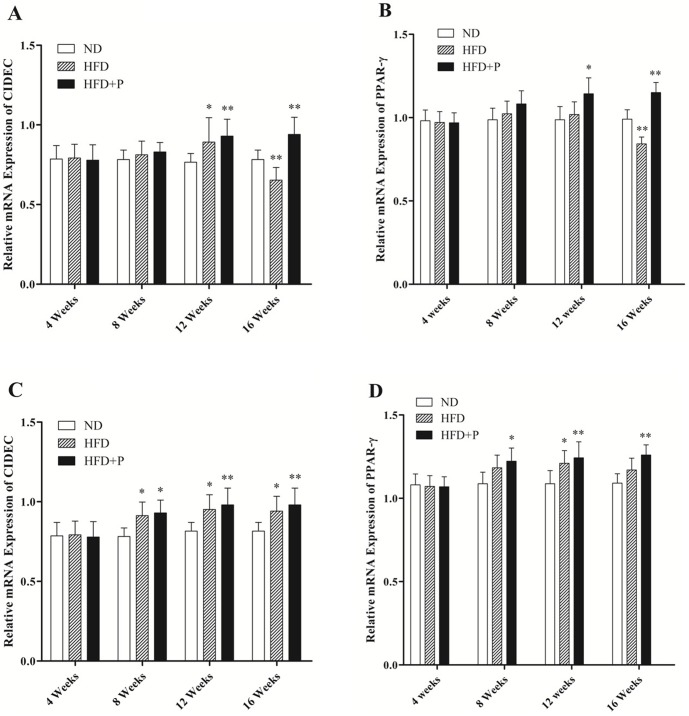
Effects of pioglitazone on mRNA expressions of CIDEC and PPAR-γ, in visceral adipose tissue (VAT) and subcutaneous adipose tissue (SAT) of mice, during different phases of obesity. Five mice from each group were sacrificed and fat tissues were collected at 4, 8, 12 and 16 weeks to evaluate the mRNA expressions of genes. Relative mRNA expressions of CIDEC in VAT (A). Relative mRNA expression of PPAR-γ in VAT (B). Relative mRNA expressions of CIDEC in SAT (C). Relative mRNA expression of PPAR-γ in SAT (D). β-actin was used as reference gene. Data was expressed as Mean±SD. *P<0.05, **P<0.01, and ***P<0.001 represent significant difference between two groups.

mRNA expression of CIDEC in VAT increased with time in HFD group during the early phase of obesity and after attaining the maximum level it decreased gradually during the late phase of obesity. The expression of PPAR-γ gene increased during the early phase of obesity in HFD group and after attaining the maximum level it remained stable until mid of late phase of obesity. Expression of PPAR-γ decreased and remained stable at a lower level in HFD group in the late phase of obesity corresponding to the increasing blood sugar. Pioglitazone significantly (P<0.05) enhanced the mRNA expressions of CIDEC and PPAR-γ in late phase of obesity (Fig.3 A–B). Interestingly, the mRNA expression of CIDEC and PPAR-γ in subcutaneous adipose tissue (SAT) increased gradually in HFD group and during the late phase of obesity this increased expression of both the genes maintained a high level and showed no significant decrease, except PPAR-γ which showed significant decrease in expression after 16 weeks of obesity. In HFD+P group the levels of CIDEC and PPAR-γ were significantly enhanced during the late phase of obesity (Fig.3 C–D).

### Immunofluorescence staining

**Figure 4:**
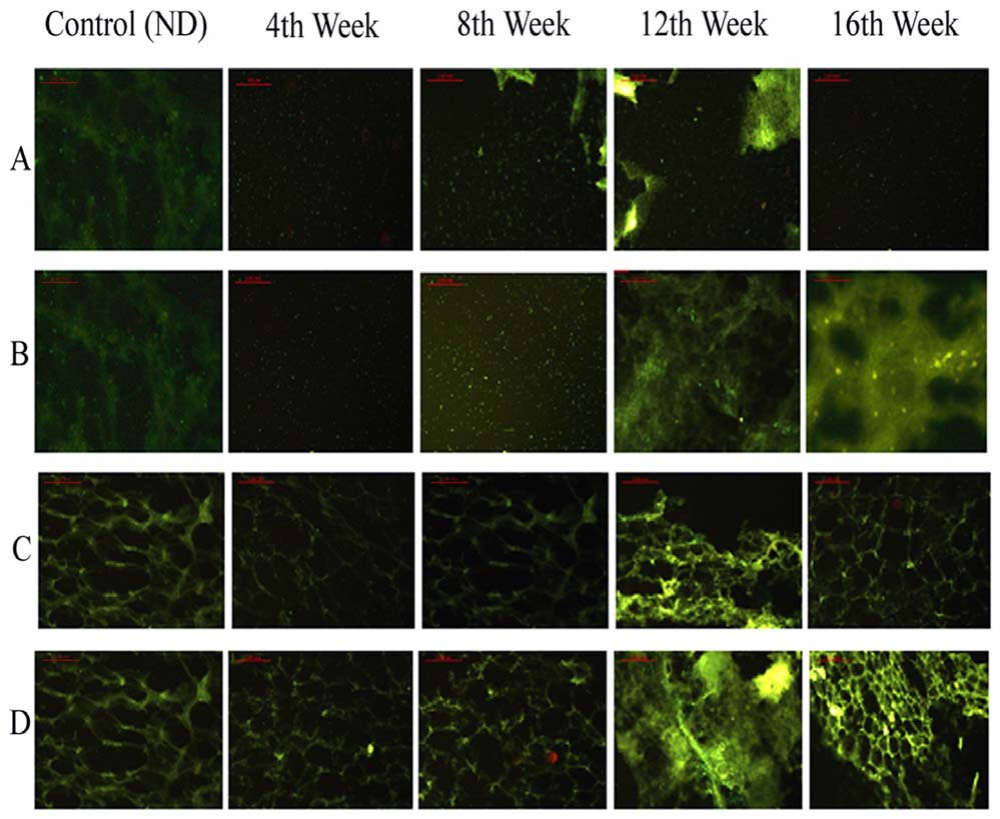
Effects of pioglitazone on expression distribution of CIDEC and PPAR-γ on VAT. Fat cells were collected at 4, 8, 12 and 16 weeks and immunofluorescence staining was used to analyze expression of CIDEC and PPAR-γ. Row A and C show expressions of PPAR-γ and CIDEC in mice fed on normal diet (ND) and 4, 8, 12 and 16 weeks of high fat diet (HFD) group. Row B and D represent effects of pioglitazone on the expressions of PPAR-γ and CIDEC in ND group, and 4, 8, 12 and 16 weeks of HFD group. Fluorescence microscope was used to observe the expressions and distribution of PPAR-γ and CIDEC.

In HFD group, the immunofluorescence staining of VAT showed that CIDEC and PPAR-γ genes expression increased in second half of early phase of obesity. In mid of late phase of obesity, expression of PPAR-γ decreased, resulting in decrease of CIDEC expression. In ND group, changes in the expression of CIDEC and PPAR-γ were not significant. The HFD+P group of mice showed increased PPAR-γ and CIDEC expressions in the early phase of obesity which remained significantly high throughout the early and late phase of obesity (Fig.4).

**Figure 5:**
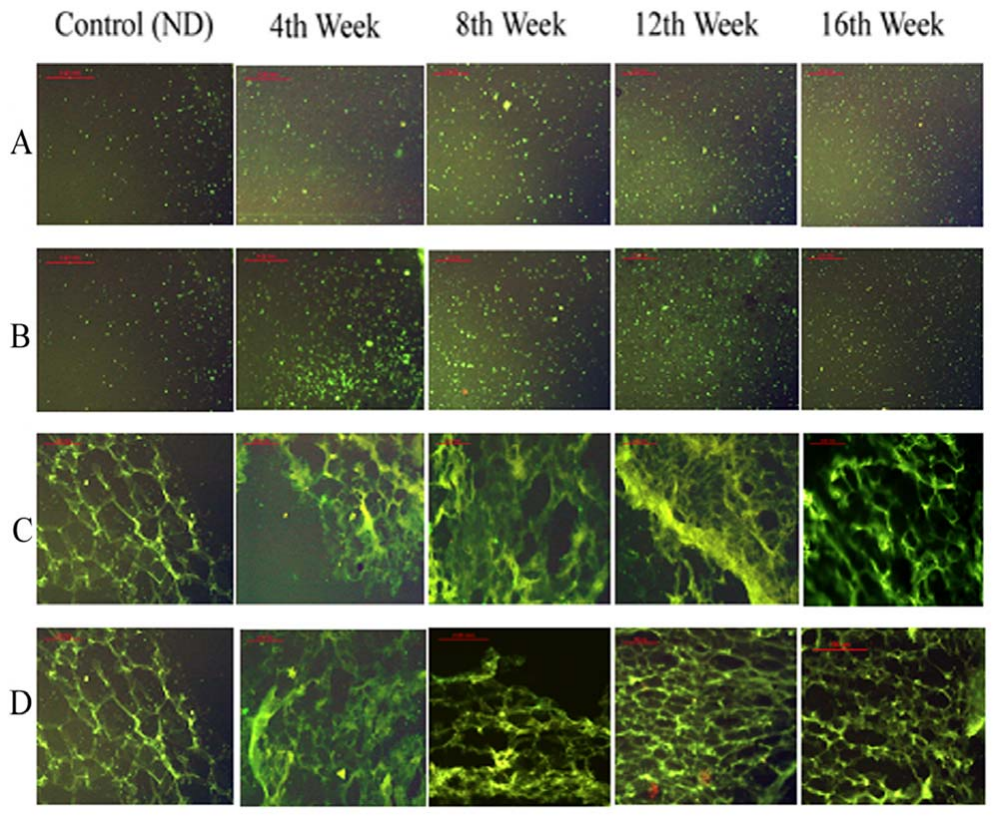
Effects of pioglitazone on expression distribution of CIDEC and PPAR-γ on SAT. Fat cells were collected at 4, 8, 12 and 16 weeks and immunofluorescence staining was used to analyze expression of CIDEC and PPAR-γ. Row A and C show expressions of PPAR-γ and CIDEC in mice fed on normal diet (ND) and 4, 8, 12 and 16 weeks of high fat diet (HFD) group. Row B and D represent effects of pioglitazone on the expressions of PPAR-γ and CIDEC in ND group, and 4, 8, 12 and 16 weeks of HFD group. Fluorescence microscope was used to observe the expressions and distribution of PPAR-γ and CIDEC.

In SAT, the genes expressions of CIDEC and PPAR-γ from HFD group increased gradually during developing obesity and in the late phase of obesity the expressions remained increased without showing significant change, while in the HFD+P group the pioglitazone significantly enhanced the expression of PPAR-γ which resulted in enhanced CIDEC expression correspondingly (Fig.5).

### Level of protein of CIDEC and PPAR-γ

**Figure 6:**
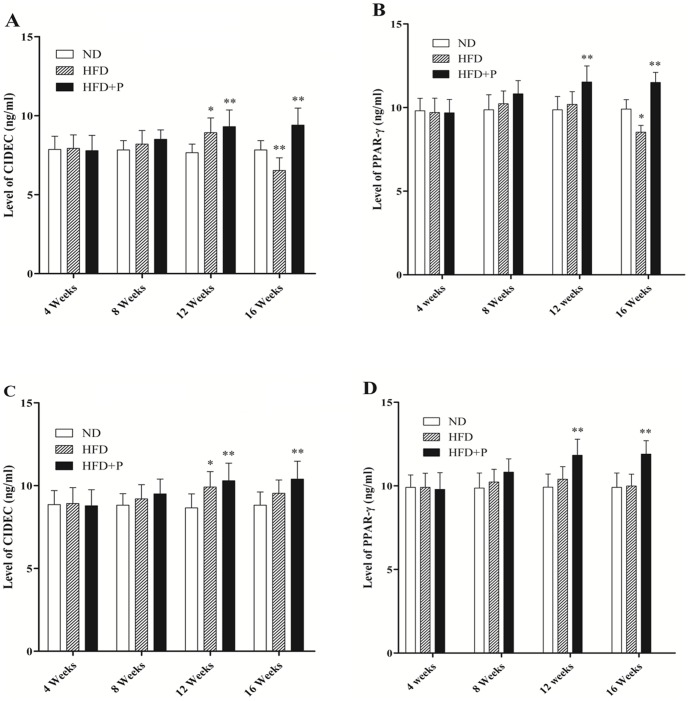
Effect of pioglitazone on level of CIDEC and PPAR-γ in VAT and SAT of mice, during different phases of obesity. Five mice from each group were sacrificed and fat tissues were collected at 4, 8, 12 and 16 weeks to evaluate the protein level of CIDEC and PPAR-γ by ELISA. Level of CIDEC in VAT (A). Level of PPAR-γ in VAT (B). Level of CIDEC in SAT (C). Level of PPAR-γ in SAT (D). Data was expressed as Mean±SD. *P<0.05, **P<0.01, and ***P<0.001 represent significant difference between two groups.

Enzyme-linked immunosorbent assay (ELISA) was used to determine the level of CIDEC and PPAR-γ in fat tissues during different phases of HFD induced obesity. We observed that the level of CIDEC and PPAR-γ enhanced gradually during early phase of obesity in visceral fat tissues of mice in HFD group but PPAR-γ showed no significant difference between ND and HFD group during early phase of obesity. Significant lowered level of CIDEC and PPAR-γ were found in late phase of obesity. Gradually enhanced levels of CIDEC and PPAR-γ were found in mice of HFD+P group when compared with ND group (Fig.6 A–B). Furthermore, the levels of CIDEC and PPAR-γ were also increased in SAT with time in HFD group during the early phase of obesity and after attaining the maximum level no significant decrease was observed during the late phase of obesity. CIDEC level was enhanced in early phase of obesity significantly, rather than level of PPAR-γ. We examined that, levels of CIDEC and PPAR-γ in mice of HFD+P group gradually increased significantly during early as well as late phase of obesity (Fig.6 B–C).

## Discussion

In present study, the effects of pioglitazone mediated sensitization of PPAR-γ were studied to understand the relationship of CIDEC in visceral and subcutaneous adipose tissue with obesity related changes including increased blood sugar level, in diet induced obesity mice. Maintained PPAR-γ prevents decrease in CIDEC gene expression during late phase of obesity hence blood sugar levels are kept in normal limits.

According to adiposity, differentiation and lipid droplets evolution, development of obesity can be divided into two major phases such as early and late phases of obesity [5], [21]. Intake of high fat diet induces increased weight gain in mice with time [22]. In early phase, pre-adipocytes differentiate into mature fat cells and the lipid droplets accumulate [23]. The volume of differentiated and matured adipocytes continues to grow, and lipid droplets further expand with rapid weight gain followed by occurrence of late phase lipolysis. We observed that the weight and body length of HFD mice increased with time and this increase was significantly high even in both phases, when compared with mice in ND group. Meanwhile the weight of mice fed on high fat diet and treated with pioglitazone, kept on increasing more than the mice in HFD group. It is already reported that pioglitazone, a drug of the class thiazolidinedione (TZD), prevents lipolysis by selectively stimulating PPAR-γ and promotes differentiation of adipocytes with lipid storage, thus is associated with increase in body weight [24]. This increased in adiposity is a key factor of weight gain in mice fed on HFD+P [25].

Visceral fat is considered as the bad fat [26]. Lipolysis occurs in VAT during the late phase of obesity with increased free fatty acids release and consequently the increase in blood sugar [27], [28]. It is reported that VAT under the influence of growth hormone participates in the regulation of insulin sensitivity [9]. A recent study confirmed that visceral fat developed in the absence of growth hormone signals contributes to increased whole body insulin sensitivity [8]. Thus removing VAT or targeted decreased effects of GH on VAT, decreases insulin resistance and hence prevents high blood sugar. Pioglitazone is highly effective oral medication for DM 2 and is also reported to control the blood sugar. It enhances insulin function, and has variable effects on serum triglyceride levels in patients with DM 2 [29]. Present study confirms that mice fed on high fat diet maintained fasting blood sugar in normal range till the mid of the late phase of obesity where blood sugar values started to increase and attained highest level during second half of late phase. The pioglitazone suppressed blood sugar level in HFD+P mice during both early and late phases of obesity. Therefore, based on previous studies and our data, we demonstrate that pioglitazone affects the VAT directly by prevention of lipolysis or indirectly by decreasing the effects of growth hormone on VAT to avoid insulin resistance hence control the blood sugar.

Adipose tissue is a major site of lipid storage and PPAR-γ is expressed in white and brown adipose tissue, the large intestine and spleen. PPAR-γ expression is highest in adipocytes and it plays a key role in the regulation of adipogenesis, energy balance, and lipid biosynthesis [30]. CIDEC is a lipid droplet-associated protein expressed in brown adipose tissues which suppresses lipolysis by stimulating the size of lipid droplets and ultimately plays role in the accumulation of triglycerides in adipocytes. PPAR-γ is essential for the transcriptional activity of CIDEC during adipogenesis [31]. It integrates and increases CIDEC expression in developing obesity [16]. When PPAR-γ is activated to a certain extent, the negative feedback mechanism inactivates its phosphorylation [32], which leads to further sustained activation of PPAR-γ, resulting in further increased CIDEC expression [33]. In the late phase of obesity, after gathering a large number of lipid droplets, begins the process of lipolysis with phosphorylation of PPAR-γ and reduction in CIDEC expression [34]. PPAR-γ agonists are reported to increase the expression of CIDEC genes in early researches [35]. It is already reported that CIDEC genes is significantly more expressed in SAT than in VAT which is true for anthropometric and clinical characteristics as well as during adipocyte differentiation [36]. VAT is responsible for insulin resistance in late obesity, during which, the genes expression of VAT is decreased more than SAT. Gene expression analysis observed through mRNA expression, protein analysis and immunofluorescence staining of VAT in HFD mice showed increased PPAR-γ and CIDEC expressions in the second half of early phase and decreased expression in the late phase of obesity, confirming the late phase phosphorylation of PPAR-γ. In HFD+P group, expression of PPAR-γ continued to increase during both, the early and late phases of obesity, showing that pioglitazone selectively stimulates PPAR-γ and interferes with its phosphorylation. CIDEC expression correspondingly kept on increasing throughout the late phase of obesity in HFD+P group mice confirming the transcriptional activation by PPAR-γ [13]. The gene expression in SAT showed increased PPAR-γ and CIDEC expressions in second half of early phase which did not show significant decrease during the late phase of obesity. The study of protein expression and distribution of PPAR-γ and CIDEC in VAT showed low expression during the late phase in HFD induced obesity and over expression in mice given high fat diet and treated with pioglitazone, which validates that VAT is responsible for the insulin resistance during the late phase of obesity, and preventing decrease in CIDEC during late phase of obesity plays an important role in controlling blood sugar levels [30].

## Conclusion

Present study demonstrates that VAT, but not SAT, is involved in the development of late obesity insulin resistance, and PPAR-γ agonist pioglitazone suppresses obesity related diabetes in mice during the early and late phases by enhancing the expressions of PPAR-γ and CIDEC and ultimately decreasing the level of blood sugar. Therefore, these findings will have significant implications concerning the genetic mechanisms involved in adipocyte differentiation, development of obesity and increased blood sugar.
